# Predictive Role of QTc Prolongation in Carbon Monoxide Poisoning-Related Delayed Neuropsychiatric Sequelae

**DOI:** 10.1155/2018/2543018

**Published:** 2018-09-25

**Authors:** Shu-Chen Liao, Yan-Chiao Mao, Yao-Min Hung, Ching-Hsing Lee, Chen-Chang Yang

**Affiliations:** ^1^Department of Emergency Medicine, Chang Gung Memorial Hospital, Keelung, Taiwan; ^2^Institute of Environmental and Occupational Health Sciences, School of Medicine, National Yang-Ming University, Taipei, Taiwan; ^3^Division of Clinical Toxicology, Department of Emergency Medicine, Taichung Veterans General Hospital, Taichung, Taiwan; ^4^Department of Emergency Medicine, Kaohsiung Veterans General Hospital, Kaohsiung, Taiwan; ^5^Division of Clinical Toxicology & Occupational Medicine, Department of Medicine, Taipei Veterans General Hospital, Taipei, Taiwan

## Abstract

**Objective:**

Delayed neuropsychiatric sequelae (DNS) are serious complications of carbon monoxide (CO) poisoning that adversely affect poisoned patients' quality of life as well as socioeconomic status. This study aimed to determine clinical predictors of DNS in patients with CO poisoning.

**Methods:**

This retrospective study included all CO-poisoned patients admitted to the emergency department (ED) of Linkou Chang Gung Memorial Hospital in Taiwan from 1 January 2009 to 31 December 2015. The medical records of all patients with CO poisoning were carefully reviewed, and relevant data were abstracted into a standardised form. Univariate and multivariate logistic regression models were used to identify predictors of DNS after CO poisoning. Receiver operating characteristic (ROC) curve analysis was used to determine the ideal cut-off value for continuous variables that predict the development of DNS.

**Results:**

A total of 760 patients with CO poisoning were identified during the study period. Among them, 466 were eligible for the analysis of predictors of DNS. In multivariate analysis, Glasgow Coma Scale <9 (odds ratio [OR], 2.74; 95% confidence interval [CI], 1.21–6.21), transient loss of consciousness (OR, 3.59; 95% CI, 1.31–9.79), longer duration from CO exposure to ED presentation (OR, 1.05; 95% CI, 1.03–1.08), and corrected QT (QTc) prolongation (OR, 2.61; 95% CI, 1.21–5.61) were found to be associated with a higher risk of DNS. The area under the ROC curve (AUC) for QTc interval measured within 6 h after exposure best predicted the development of DNS, with a result of 0.729 (95% CI 0.660–0.791). Moreover, the best cut-off value of the QTc interval was 471 ms, with a sensitivity of 53.3% and a specificity of 85.1%.

**Conclusions:**

We identified several potential predictors of DNS following CO poisoning. Among them, QTc prolongation found within 6 h after exposure is a novel predictor of DNS, which may be helpful in the future care of patients with CO poisoning.

## 1. Introduction

Carbon monoxide (CO) is a colourless, odourless, and highly toxic gas that is produced during incomplete carbon combustion. CO poisoning can occur following the inhalation of partially combusted charcoal briquettes, fuel gas, or oil in a poorly ventilated place. Domestic exposure can occur through incomplete combustion in gas-furnace water heaters, through inhalation of exhaust gas from automobiles, or in fire accidents [1, 2].

Since 2002, CO poisoning has been one of the leading causes of death by suicide in Taiwan. According to the National Suicide Database of Taiwan, the number of deaths by suicide using ‘gases in domestic use' or ‘other gases and vapours' has risen year-by-year, peaking in 2006. The number of deaths from burning charcoal has declined since 2012 after the government enacted regulation on the sale of charcoal (http://dep.mohw.gov.tw/DOMHAOH/cp-332-8883-107.html).

Most patients with CO poisoning present to the emergency department (ED) with nonspecific symptoms such as headache, dizziness, malaise, asthenia, nausea, vomiting, flu-like syndrome, transient loss of consciousness, altered mental status, chest tightness, palpitation, and dyspnoea. Life-threatening cardiac arrhythmia, seizures, and coma may also develop in cases of severe CO poisoning. In the absence of a clear medical history, the aforementioned manifestations may create diagnostic challenges for emergency physicians [3, 4].

In addition to causing acute morbidity and mortality, CO poisoning can cause delayed neuropsychiatric sequelae (DNS) that occur days or weeks after initial complete clinical recovery from acute poisoning [5]. The mechanisms underlying DNS remain unclear; however, progressive inflammation may be involved. CO diffuses quickly into the blood via the lungs and causes direct tissue hypoxaemia through the formation of carboxyhaemoglobin (COHb) and a leftward shift of the oxyhaemoglobin dissociation curve. CO also binds to haeme-containing proteins such as cytochrome *c* oxidase and platelet haemoprotein. All the aforementioned mechanisms can induce a chain reaction of inflammation that increases the production of reactive oxygen species and induces oxidative stress, lipid peroxidation, and apoptosis [5–7].

The incidence of DNS following CO poisoning varies widely from 3% to 46%, and the lag time ranges between 2 days and 6 weeks after exposure [8–11]. Because of the lack of standard diagnostic criteria, most reported DNS include a broad spectrum of neurological deficits, cognitive impairments, and psychological disorders** (Table 1)** [3]. In patients with CO poisoning, the occurrence of DNS can result in decreased ability to conduct instrumental as well as other activities of daily living [12]. Thus, the prompt identification of CO-poisoned patients who are at risk of DNS is vital to ensure that effective treatment is provided to reduce the risk of DNS.

Studies have revealed various clinical predictors of DNS following CO poisoning, including age >36 years, long exposure to CO, higher COHb levels (>25%), damage in the globus pallidus or white matter at initial ED presentation, Glasgow Coma Scale (GCS) score <9, seizures, systolic blood pressure <90 mmHg at ED presentation, elevated creatine phosphokinase concentration, leukocytosis, and a positive Babinski reflex [1, 3, 4, 8–14]. However, these findings remain inconclusive.

In acute CO poisoning, numerous electrocardiogram (ECG) alterations, such as P-wave dispersion, corrected QT (QTc) dispersion, and QTc prolongation, have been reported [2, 15–18], which might be related to life-threatening arrhythmias. The possible mechanism of ECG alterations in CO poisoning is related to S-nitrosylation of the Na^+^ channel, which increases the late inward Na^+^ current, resulting in prolongation of the action potential and the associated intracellular Ca^2+^ transients, subsequently causing disruption of repolarisation and prolongation of the QT interval. However, no study has determined the relation between acute ECG alterations and DNS [19]. To elucidate the predictive role of ECG alterations, particularly QTc prolongation, and to identify other clinical predictors of CO-related DNS, we conducted a retrospective cohort study of acute CO-poisoned patients in a tertiary medical center in Taiwan.

## 2. Methods

### 2.1. Ethics

The study protocol was approved by the Institutional Review Board of Chang Gung Medical Foundation (104-7628C) and was granted permission by the Medical Ethics Committee of Chang Gung Memorial Hospital.

### 2.2. Study Design, Study Population, and Participant Selection

A retrospective medical record review was conducted for all CO-poisoned patients (ICD-9-CM code as 986) admitted to the ED of Linkou Chang Gung Memorial Hospital from 1 January 2009 to 31 December 2015. COHb levels were measured using an arterial blood gas analyser with a CO oximeter. Patients enrolled into this study were required to meet at least one of the following criteria: (1) a COHb level of >5% in nonsmokers and >10% in smokers at presentation and (2) delayed presentation with an unambiguous history of CO poisoning, such as being found in a confined space with burning charcoal, exposure to car or machine exhaust, or living in a place with a faulty water heater. The medical records of all patients were reviewed, and information on clinical symptoms and laboratory findings on admission to the ED, circumstances of exposure, treatment received, clinical outcomes, and other information available at the ED were abstracted.

### 2.3. Data Collection and Definition of Variables

All patients were provided with 100% oxygen from a “nonrebreathing” facemask (NRM) as soon as CO poisoning was suspected at the ED or prehospital setting when attended by emergency services. Information on the following variables was collected on admission to the ED: age; sex; psychiatric history; referral institution; intentional or accidental exposure; CO exposure source; vital signs; Taiwan Triage and Acuity Scale (TTAS); concomitant use of tranquilisers; transient loss of consciousness; duration of loss of consciousness; duration from CO exposure to ED admission; evidence of myocardial injury (defined as electrocardiographic signs of ischaemia/arrhythmias and cardiac enzyme elevation); ECG; images such as brain computed tomography (CT); treatment modality (hyperbaric oxygen therapy(HBOT) at 2.5 atmosphere absolute for 90 minutes/session or 100% normobaric oxygen therapy through NRM); number of sessions of HBOT; duration from CO exposure to HBOT administration; and length of hospital and intensive care unit stay. The results of blood tests on ED visit and admission, including complete blood cell count, arterial blood gas analysis, COHb level, troponin I, creatine phosphokinase, and serum alcohol level, were also collected. Data on urine benzodiazepine and illicit drug screening were abstracted as well.

ECG findings were further analysed to calculate the QTc interval. The QTc interval is the measured QT interval corrected for heart rate using Bazett's formula (QT/RRK). QTc prolongation has been defined as follows: longer than 440 ms in men and longer than 460 ms in women [2, 20].

All CO-poisoned patients received 100% oxygen supplement through NRM upon ED arrival. Some patients received HBOT after consultation with an HBOT specialist following the management guideline of CO poisoning of the American Undersea and Hyperbaric Medical Society, 13th Edition (https://www.uhms.org/carbon-monoxide-poisoning/carbon-monoxide-poisoning.html).

DNS were defined as the recurrence of original symptoms or the development of new symptoms such as difficulty concentrating, lethargy, emotional lability, mutism, amnestic syndromes, dementia, psychomotor retardation, Parkinsonism, apraxia, unsteady gait, and urinary incontinence, within 2-42 days after CO poisoning [3, 5, 6, 10–12, 14]. All poisoned patients were invited to the follow-up visits from hospital discharge in outpatient clinic. A comprehensive neurological exam was performed and also the mental status examination during the visit. The follow-up duration to observe the development of DNS was at least 6 months.

### 2.4. Statistical Analysis

Demographic and clinical data of the patients with and without DNS were compared using Fisher's exact test for categorical variables or the Mann–Whitney *U* test for continuous variables because most continuous variables were not normally distributed. To investigate the factors associated with DNS development, we introduced the variables with* P *< 0.2 in univariate analyses into a multivariable logistic regression model with stepwise selection. The ability of QTc to predict DNS development was evaluated using receiver operating characteristic (ROC) curve analysis. Finally, the proportion of QTc prolongation in patients with and without DNS stratified by duration from CO exposure to ECG recording was compared using Fisher's exact test. Data analysis was conducted using MedCalc Statistical Software version 13.1.2 (MedCalc Software bvba, Ostend, Belgium; http://www.medcalc.org; 2014). All odds ratios (ORs) were reported with relevant 95% confidence intervals (CIs).* P* < 0.05 was considered statistically significant.

## 3. Results

A total of 760 patients with CO poisoning who met the inclusion criteria of this study were admitted to the ED of Linkou Chang Gung Memorial Hospital from 2009 to 2015. Among them, 466 patients were eligible for further DNS detection** (Supplementary Table **1**)**. A total of 294 patients were excluded because we were unable to confirm or exclude the diagnosis of DNS. Among the excluded patients, 19 were in a persistent vegetative state or unresponsive wakefulness; 12 died during ED admission; 3 were discharged from ED in a moribund status upon family's request; and contact was lost with 260 after discharge from the ED.

Among the 466 included patients, almost half were men (230/466, 49.4%), and the median age was 33 years (interquartile: 22–45 years). A total of 223 patients (47.9%) were transferred to our ED from another medical institution; 190 patients (40.8%) had been attempting suicide. Inappropriately ventilated gas from heating appliances was the most common cause of CO poisoning (238/466, 51.1%), followed by charcoal burning (196/466, 42.1%). Most patients presented with clear consciousness upon arrival, and 62 (62/466, 13.3%) had poor consciousness with a GCS of <9. More than half had experienced transient loss of consciousness after CO poisoning (292/466, 62.7%), and most patients presented with loss of consciousness for <6 h (210/292, 71.9%). The median initial COHb level upon admission was 10.1% (median: 10.1%, interquartile: 4.0%–22.6%), and slightly over half of patients had leukocytosis (245/444, 56.5%). Laboratory test results revealed that 64 (18%) patients had myocardial injury. The median time from CO exposure to ED admission and to ECG recording was 4.0 and 5.1 h, respectively. A 12-lead ECG revealed that 42.1% of patients had QTc prolongation. A total of 279 (59.9%) received HBOT, with a median total of 3 sessions (**Table 2**).

### 3.1. Univariate Analysis of Potential Risk Factors for DNS

Among the 466 patients with adequate follow-up period to detect DNS, DNS diagnosis was confirmed in 62 (13.3%), with a median duration from CO exposure of 10 days (interquartile: 7–24 days). Univariate analysis identified the following risk factors for DNS: old age (*P* = 0.005); psychiatric history (*P* = 0.047); poor consciousness level at ED presentation and GCS <9 at admission (*P* < 0.001); higher TTAS (*P* = 0.014); intentional poisoning (*P* < 0.001); charcoal burning (*P* < 0.001); concomitant tranquiliser use (*P* = 0.015); transient loss of consciousness and longer duration of loss of consciousness (*P* < 0.001); longer duration from CO exposure to ED admission; and ECG revealing QTc prolongation. Laboratory test results revealed that the presence of myocardial injury (Trop-I > 0.5 ng/mL) and leukocytosis (WBC > 10.000/mL) was also associated with DNS (*P *< 0.001 and* P *= 0.004, respectively) (**Table 2**).

### 3.2. Multivariable Logistic Regression of Risk Factors for DNS

Variables with* P* < 0.2 in univariate analyses (as shown in Table 2) were introduced into the multivariable model using stepwise selection. The results demonstrated that GCS <9 (OR, 2.74; 95% CI, 1.21–6.21), transient loss of consciousness (OR, 3.59; 95% CI, 1.31–9.79), longer duration from exposure to ED admission (OR, 1.05; 95% CI, 1.03–1.08), and QTc prolongation (OR, 2.61; 95% CI, 1.21–5.61) were associated with a higher risk of DNS. In addition, there was a trend indicating that increased age might be correlated with DNS development (OR, 1.18;* P* = 0.089) (**Table 3**).

**Figure 1:**
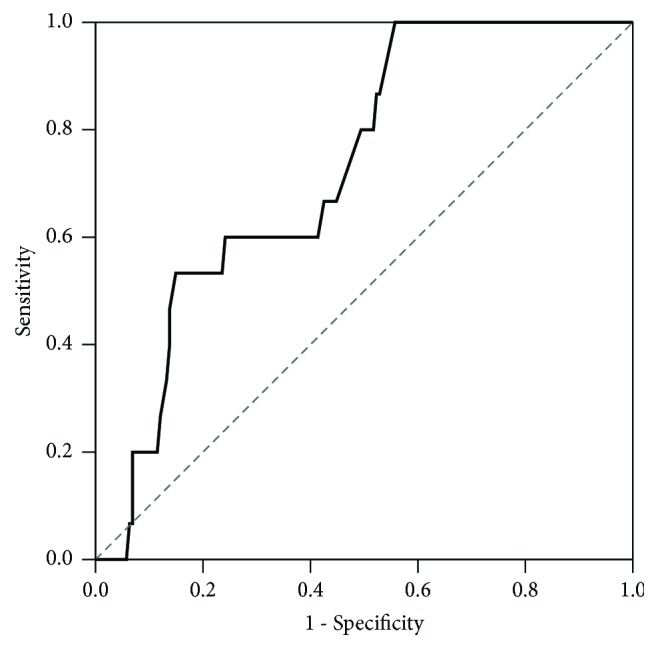
ROC curve of QTc for discriminating DNS by ECG recording performed within 6 hours after CO exposure. The area under ROC (AUC) was 0.729 (95% CI: 0.660–0.791). The best cut-off point was 471 ms with a sensitivity of 53.3%, a specificity of 85.1%, a positive predictive value of 23.5%, and a negative predictive value of 95.5%.

**Figure 2:**
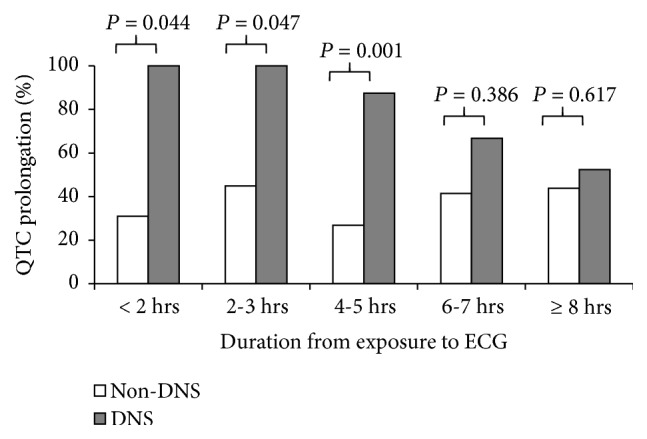
QTc prolongation in patients with and without DNS stratified by duration from CO exposure to ECG recording.

**Table 4** displays summaries of the ROC curves for the discrimination ability of QTc for DNS development stratified by duration from CO exposure to ECG recording. The result revealed that the discrimination ability was significant and acceptable for durations <2 h (AUC = 0.747), 2–3 h (AUC = 0.740), and 4–5 h (AUC = 0.743). The discrimination ability was poorer for durations of 6–7 h and ≥8 h.**Figure 1** plots the ROC curve of QTc for discriminating DNS within 6 h from exposure to ECG recording. The relevant AUC was 0.729 (95% CI, 0.660–0.791), and the best cut-off point was 471 ms, with a sensitivity of 53.3% and a specificity of 85.1%, a positive predictive value of 23.5%, and a negative predictive value of 95.5%. Similarly, we found that the proportion of QTc prolongation in patients with and without DNS was statistically significant in periods <2 h (100% versus 31%,* P* = 0.044), 2–3 h (100% versus 44.9%,* P* = 0.047), and 4–5 h (87.5% versus 26.9%,* P* = 0.001) from CO exposure to ECG recording (**Figure 2**).

## 4. Discussion

Neurological deficits and cognitive disorders impede the ability of patients to exercise self-care as well to participate in occupational and social activities; therefore, such conditions can affect the lives of not only patients but also their families [4]. Several predictors of DNS following CO poisoning have been proposed: GCS <9 [3], MMSE scores, positive findings on brain CT images [10], transient loss of consciousness, age >36 years, COHb levels ≥25% [5], serious conscious disturbance at ED presentation, elevated creatine kinase levels, elevated creatine kinase-MB and lactate dehydrogenase levels, and low Global Assessment Scale scores [1].

This study aimed to identify predictors of DNS after CO poisoning, which may be of use to emergency physicians. Furthermore, to the best of our knowledge, this retrospective study enrolled the largest number of patients to date for any such study.

The severity of CO poisoning is determined by the duration of CO exposure and the concentration of CO in the atmosphere, but not by the COHb level at admission [1, 3, 21–23]. COHb has a fundamental diagnostic role, but the lack of its prognostic value has been confirmed by many studies. COHb levels are influenced by several factors, such as prehospital oxygen administration and time elapsed between exposure cessation and hospital admission [3].

In this study, we determined that GCS <9, transient loss of consciousness, longer duration from CO exposure to ED admission, and ECG revealing QTc prolongation were independent predictors of DNS in CO-poisoned patients (Table 3). Among these factors, other studies have reported lower GCS and transient loss of consciousness as predictors of DNS in CO-poisoned patients [3, 8, 10], but this study provides the first indication of longer duration from CO exposure to ED admission and ECG revealing QTc prolongation as predictors. This finding may help emergency physicians make prompt diagnoses of DNS and arrange further treatment for patients with CO poisoning. For patients at a high risk of DNS, intensive treatment and monitoring, including earlier HBOT and follow-up for neuropsychiatric sequelae, should be considered.

A longer duration from CO exposure to ED admission indicates prolonged CO exposure or a longer interval from exposure cessation to hospital treatment. Longer exposure to CO is one of the determinants of severity, and a longer interval from CO exposure to ED admission implies a delay in treatment with any type of oxygen supplement such as normobaric oxygen or HBOT.

Acute CO poisoning frequently affects the repolarisation of myocardium electrophysiology, resulting in abnormal ECG findings such as QTc prolongation and QTc dispersion [16, 18, 19, 24]. These changes may lead to life-threatening cardiac arrhythmia or proarrhythmia effects [19]. Ventricular repolarisation can be evaluated by measuring the QT interval, QTc interval, and QTc dispersion. In* in vitro* studies, CO poisoning resulted in elevated NO levels in myocytes, causing S-nitrosylation of the Na^+^ channel and increasing the late component of the inward Na^+^ current. This subsequently resulted in prolongation of the action potential and the associated intracellular Ca2^+^ transients, leading to disruption of ventricular myocyte repolarisation and QT prolongation [2, 15]. Studies have also suggested that prolonged QTc and QTc dispersion increased the risk of atrial and ventricular arrhythmia in patients with CO poisoning [2, 16]. However, no study has linked QTc interval or QTc dispersion with the prediction of DNS in patients with CO poisoning. Theoretically, the more severe a case of CO poisoning is, the higher the chance of DNS development. However, to the best of our knowledge, few biomarkers or poisoning severity scores are available that can be used as a pragmatic surrogate for CO poisoning severity in the ED setting. Based on the findings of the present study, QTc prolongation may be more representative of CO poisoning severity than previously thought.

QTc interval and its dispersion were dynamic parameters for CO poisoning in the present study. QTc prolongation was found at admission and resolved significantly between 6 and 24 h after admission [2, 17]. Our data also indicated that conducting ECG within 6 h of CO exposure had a discernible effect on the prediction of DNS, with an AUC of 0.729 (95% CI: 0.660–0.791) and a best cut-off point of 471 ms of the QTc interval (Figure 1). However, no statistical significance was revealed for ECG conducted >6 h after CO exposure (Figure 2). This is reasonable because CO is eliminated through pulmonary circulation, and the elimination half-life of CO is approximately 300, 90, and 30 min under the breathing of room air, 100% oxygen via a reservoir or NRM facemask, and HBOT, respectively [9]. In our study, the median time from CO exposure to ED admission was 4 h (interquartile: 2.5–8 h). Nevertheless, all CO-poisoned patients had received prehospital normobaric oxygen therapy, which could lead to pulmonary decontamination of CO and subsequently confounded the results of ECG.

This study has several limitations. First of all, there has been a steady decline in the number of follow-up visits after discharging a patient from the ED, which has resulted in a decreased ability to detect DNS and might have adversely affected the statistical power of this study. Secondly, we adopted a symptom-based diagnostic criteria of DNS because of the lack of standard ones. Previous studies employed bundle battery of test [5], Folstein Mini-Mental Status Examination (MMSE) [10], Hamilton Depression Rating Scale (HDRS), Beck Anxiety Inventory, Wechsler Memory Scale-Revised (WMS-R), and Verbal Memory Process Test (VMPT) [4] as the diagnostic criteria of DNS, but these tests were still inconclusive. Thirdly, if we could obtain serial ECG follow-ups in every single patient, we wound be able to detect dynamic QTc duration and provide more strong evidence on the usefulness of QTc prolongation in predicting DNS following CO poisoning. Finally, since this was a retrospective study and the data were abstracted from a chart review, the clinical presentations or medical records might not have been completely documented, which could then lead to the loss of statistical significance in certain potential predictors of DNS.

## 5. Conclusion

DNS is the most serious complication after patient recovery from acute CO poisoning. DNS represents a challenge to emergency physicians because no established criteria exist for determining the risk of DNS in the ED setting. We conducted a retrospective study to identify independent predictors of DNS after CO poisoning. The results revealed that GCS <9, transient loss of consciousness, longer duration from CO exposure to ED presentation, and ECG revealing QTc prolongation are independent predictors for DNS of CO poisoning. Of these factors, ECG revealing QTc prolongation within 6 hours after CO exposure is first identified as a predictor of DNS. Early detection of CO-poisoned patients with a high DNS risk at the ED may help physicians plan better therapeutic strategies and arrange suitable follow-up treatment for the patients.

## Figures and Tables

**Table 1:** Previously reported signs and symptoms of DNS [3].

**Neurological sequelae**	**Cognitive and psychological sequelae**
Parkinson-like syndromes	Concentration deficit
Gait and motor disturbances	Memory loss
Bradykinesia	Cognitive impairment
Intention tremor	Dementia
Myoclonus	Personality changes
Dyspraxia	Anxiety
Dysphasia	Extreme emotional lability
Ataxia	Psychosis
Postural instability	Depression
Vertigo	Mania
Cortical blindness	Insomnia
Hearing loss, tinnitus	
Chorea	
EEG abnormalities	
Epilepsy	
Peripheral neuropathies	
Recurrent headache	
Fecal/urinary incontinence	

**Table 2:** Demographic and clinical data of patients with and without DNS.

Variables	Total (*N* = 466)	DNS (*n* = 62)	Non-DNS (*n* = 404)	*P *value
Male gender	230 (49.4)	35 (56.5)	195 (48.3)	0.275
Age (years)	33 [22, 45]	41 [26, 52]	32 [21, 43]	0.005
Psychiatric history	82 (17.6)	17 (27.4)	65 (16.1)	0.047
Pulse, beats/min	100 [84, 114]	96 [84, 110]	100 [85, 115]	0.346
Pulse > 100 beats/min	216 (47.7)	25 (41.0)	191 (48.7)	0.273
Glasgow Coma Score (GCS)	15 [14, 15]	14 [7, 15]	15 [15, 15]	<0.001
GCS less than 9	62 (13.3)	19 (30.6)	43 (10.6)	<0.001
Triage scale				0.014
1, resuscitation	74 (15.9)	18 (29.0)	56 (13.9)	
2, emergent	290 (62.2)	33 (53.2)	257 (63.6)	
3, urgent	94 (20.2)	9 (14.5)	85 (21.0)	
4/5 less urgent/not urgent	8 (1.7)	2 (3.2)	6 (1.5)	
Transferred from outside institution	223 (47.9)	37 (59.7)	186 (46.0)	0.056
Attempted suicide	190 (40.8)	42 (67.7)	148 (36.6)	<0.001
Source of CO poisoning				<0.001
Charcoal burning	196 (42.1)	45 (72.6)	151 (37.4)	
Inappropriately ventilated gas heating appliances	238 (51.1)	13 (21.0)	225 (55.7)	
Others	32 (6.9)	4 (6.5)	28 (6.9)	
Concomitant use with tranquilizer	63 (13.5)	15 (24.2)	48 (11.9)	0.015
Transient loss of consciousness	292 (62.7)	55 (88.7)	237 (58.7)	<0.001
Duration of loss of consciousness				<0.001
< 6 hours	210 (71.9)	25 (45.5)	185 (78.1)	
6-12 hours	28 (9.6)	5 (9.1)	23 (9.7)	
13-24 hours	20 (6.8)	13 (23.6)	7 (3.0)	
> 24 hours	16 (5.5)	10 (18.2)	6 (2.5)	
> 48 hours	18 (6.2)	2 (3.6)	16 (6.8)	
Leukocytosis	245 (56.5)	43 (74.1)	202 (53.7)	0.004
Metabolic acidosis	67 (19.1)	9 (19.6)	58 (19.1)	1.000
COHb, %	10.1 [4.0, 22.6]	7.4 [3.1, 19.8]	10.9 [4.2, 22.7]	0.095
Troponin I, ng/mL	0.20 [0.01, 1.83]	0.93 [0.15, 4.30]	0.14 [0.01, 1.72]	0.001
Evidence of myocardial injury	64 (18.0)	21 (42.9)	43 (14.0)	<0.001
Time from CO exposure to ED (hours)	4.0 [2.5, 8.0]	9.8 [4.8, 21.8]	4.0 [2.5, 7.0]	<0.001
Time from CO exposure to ECG recording (hours)	5.1 [3.1, 8.5]	8.3 [4.5, 21.1]	4.8 [3.0, 7.5]	<0.001
QTc prolongation	149 (42.1)	37 (69.8)	112 (37.2)	<0.001
Brain CT imaging study at first medical institution	131 (28.1)	40 (64.5)	91 (22.5)	<0.001
HBOT	279 (59.9)	47 (75.8)	232 (57.4)	0.008
Number of HBOT sessions	3 [1, 3]	4 [2, 6]	2 [1, 3]	<0.001
Length of hospital stay (days)	1 [0, 3]	5 [1, 11]	1 [0, 3]	<0.001
ICU stay (days)	26 (5.6)	10 (16.1)	16 (4.0)	0.001
Lucid interval, duration from exposure to DNS (days)	NA	10 [7, 24]	NA	NA

Continuous data were expressed as median (25^th^ and 75^th^ percentiles); categorical data were presented as frequency (proportion).

**Table 3:** Factors associated with DNS development in multivariate logistic regression analysis.

Variables	Odds ratio (95% CI)	*P* value
Age (per 10 years)	1.18 (0.98–1.42)	0.089
GCS < 9	2.74 (1.21–6.21)	0.016
Transient loss of consciousness	3.59 (1.31–9.79)	0.013
Duration from CO exposure to ED (hours)	1.05 (1.03–1.08)	<0.001
QTc prolongation		
Yes vs. No	2.61 (1.21–5.61)	0.014
Yes vs. Unknown	0.77 (0.24–2.45)	0.653

CI, confidence interval.

**Table 4:** ROC curves of QTc for discriminating DNS stratified by duration from CO exposure to ECG recording.

Hour from exposure to ECG recording	AUC	95% Confidence Interval of AUC	*P* value
< 2hrs.	0.747	0.563 to 0.883	0.036
2-3 hrs.	0.740	0.632 to 0.831	0.034
4-5 hrs.	0.743	0.630 to 0.837	0.007
6-7 hrs.	0.683	0.531 to 0.811	0.221
≥ 8 hrs.	0.518	0.408 to 0.626	0.821

AUC, area under the curve.

## Data Availability

The data used to support the findings of this study are restricted by the Chang Gung Medical Foundation Institutional Review Board in order to protect patient privacy. Data are available from the corresponding author: Chen-Chang Yang for researchers who meet the criteria for access to confidential data.

## Supplementary Materials

Supplementary Materials Supplement Table 1. Demographic and clinical data of patients with and without DNS detection. Supplement Table 1 lists the demographic and clinical data of patients with and without DNS detection. Compared to patients without DNS detection, those with DNS detection had the following characteristics: higher proportion of pulse > 100 beat/min, higher proportion of GCS less than 9, lower score of Triage scale, less likely to be transferred from outside institution, higher proportion of evidence of myocardial injury, higher proportion of brain CT at first medical institution, less likely to receive HBO2 therapy, receiving a greater number of HBO2 sessions, having longer length of hospital stay, and more likely to stay in ICU. Supplement Table 2. Demographic and clinical data of patients with and without electrocardiography (ECG). Supplement Table 2 lists the demographic and clinical data of patients with and without ECG. Compared to patients without ECG, those with ECG detection had the following characteristics: less likely to be male, being older, lower proportion of pulse > 100 beat/min, higher proportion of leukocytosis, higher proportion of evidence of myocardial injury, and more likely to have QTc prolongation.
