# Supplementary material for: Predictive Role of QTc Prolongation in Carbon Monoxide Poisoning-Related Delayed Neuropsychiatric Sequelae
Source: Biomed Res Int. 2018 Sep 25;2018:2543018. doi: 10.1155/2018/2543018 (PMC6176310; doi:10.1155/2018/2543018)
Supplement: Supplementary Materials — Supplement Table 1. Demographic and clinical data of patients with and without DNS detection. Supplement Table 1 lists the demographic and clinical data of patients with and without DNS detection. Compared to patients without DNS detection, those with DNS detection had the following characteristics: higher proportion of pulse > 100 beat/min, higher proportion of GCS less than 9, lower score of Triage scale, less likely to be transferred from outside institution, higher proportion of evidence of myocardial injury, higher proportion of brain CT at first medical institution, less likely to receive HBO2 therapy, receiving a greater number of HBO2 sessions, having longer length of hospital stay, and more likely to stay in ICU. Supplement Table 2. Demographic and clinical data of patients with and without electrocardiography (ECG). Supplement Table 2 lists the demographic and clinical data of patients with and without ECG. Compared to patients without ECG, those with ECG detection had the following characteristics: less likely to be male, being older, lower proportion of pulse > 100 beat/min, higher proportion of leukocytosis, higher proportion of evidence of myocardial injury, and more likely to have QTc prolongation. [file 2543018.f1.doc]

**Supplement Table 1**. Demographic and clinical data of patients with and without data available for DNS detection

Supplement Table 1 lists the demographic and clinical data of patients with and without data available for DNS detection. Compared to patients without data for DNS detection, those with data for DNS detection had the following characteristics: higher proportion of pulse > 100 beats/min, higher proportion of GCS less than 9, lower score of Triage scale, less likely to be transferred from outside institution, higher proportion of evidence of myocardial injury, higher proportion of receiving brain CT imaging study at first medical institution, less likely to receive HBOT, received a greater number of HBOT sessions, had longer length of hospital stay and more likely to stay in ICU.

**Supplement Table 2**. Demographic and clinical data of patients with and without electrocardiography (ECG)

Supplement Table 2 lists the demographic and clinical data of patients with and without ECG. Compared to patients without ECG, those with ECG recording had the following characteristics: less likely to be male, older, lower proportion of pulse > 100 beats/min, higher proportion of leukocytosis, higher proportion of evidence of myocardial injury and more likely to have QTc prolongation.

**Supplementary Table 1**. Demographic and clinical data of patients with and without data available for DNS detection

| Variables | Total  (*N* = 760) | With data available for DNS detection  (*n* = 466) | Without data available for DNS detection (*n* = 294) | *P* value |
| --- | --- | --- | --- | --- |
| Male gender | 373 (49.1) | 230 (49.4) | 143 (48.6) | 0.847 |
| Age (years) | 32 [22, 43] | 33 [22, 45] | 31 [22, 41] | 0.223 |
| Psychiatric history | 133 (17.5) | 82 (17.6) | 51 (17.3) | 0.930 |
| Pulse, beats/min | 98 [83, 111] | 100 [84, 114] | 96 [81, 108] | 0.005 |
| Pulse > 100 beats/min | 338 (44.5) | 216 (46.4) | 122 (41.5) | 0.013 |
| Glasgow Coma Score (GCS) | 15 [15, 15] | 15 [14, 15] | 15 [15, 15] | 0.002 |
| GCS less than 9 | 85 (11.2) | 62 (13.3) | 23 (7.8) | 0.020 |
| Triage scale |  |  |  | 0.002 |
| 1, resuscitation | 97 (12.8) | 74 (15.9) | 23 (7.8) |  |
| 2, emergent | 487 (64.1) | 290 (62.2) | 197 (67.0) |  |
| 3, urgent | 158 (20.8) | 94 (20.2) | 64 (21.8) |  |
| 4, less urgent | 11 (1.4) | 8 (1.7) | 3 (1.0) |  |
| 5, not urgent | 1 (0.1) | 0 (0.0) | 1 (0.3) |  |
| Transferred from outside institution | 420 (55.3) | 223 (47.9) | 197 (67.0) | <0.001 |
| Attempted suicide | 307 (40.4) | 190 (40.8) | 117 (39.8) | 0.789 |
| Source of CO poisoning |  |  |  | 0.569 |
| Charcoal burning | 314 (41.3) | 196 (42.1) | 118 (40.1) |  |
| Water heater incomplete combustion or incorrect use of furnace | 398 (52.4) | 238 (51.1) | 160 (54.4) |  |
| Others | 48 (6.3) | 32 (6.9) | 16 (5.4) |  |
| Concomitant use with tranquilizer | 100 (13.2) | 63 (13.5) | 37 (12.6) | 0.711 |
| Transient loss of consciousness | 460 (60.5) | 292 (62.7) | 168 (57.1) | 0.130 |
| Duration of loss of consciousness |  |  |  | 0.002 |
| < 6 hours | 347 (45.7) | 210 (45.1) | 137 (46.6) |  |
| 6-12 hours | 49 (6.4) | 28 (6.0) | 21 (7.1) |  |
| 13-24 hours | 27 (3.6) | 20 (4.3) | 7 (2.4) |  |
| > 24 hours | 16 (2.1) | 16 (3.4) | 1. (0.0) |  |
| > 48 hours | 21 (2.8) | 18 (3.9) | 3 (1.0) |  |
| Leukocytosis | 380 (50.0) | 245 (52.6) | 135 (45.9) | 0.069 |
| Metabolic acidosis | 100 (13.2) | 67 (14.4) | 33 (11.2) | 0.435 |
| COHb, % | 9.6 [4.1, 21.1] | 10.1 [4.0, 22.6] | 9.2 [4.1, 17.4] | 0.138 |
| Troponin I, ng/mL | 0.16 [0.01, 1.80] | 0.20 [0.01, 1.83] | 0.10 [0.01, 1.80] | 0.132 |
| Evidence of myocardial injury | 87 (11.4) | 64 (13.7) | 23 (7.8) | 0.030 |
| Time from CO exposure to ED (hours) | 4.5 [3.0, 8.0] | 4.0 [2.5, 8.0] | 5.0 [3.5, 7.5] | 0.169 |
| Time from CO exposure to ECG (hours) | 5.3 [3.4, 8.2] | 5.1 [3.1, 8.5] | 5.5 [3.8, 8.1] | 0.207 |
| QTc prolongation | 231 (30.4) | 149 (32.0) | 82 (27.9) | 0.491 |
| Brain CT imaging study at first medical institution | 131 (17.2) | 131 (28.1) | 0 (0.0) | <0.001 |
| HBOT | 478 (62.9) | 279 (59.9) | 199 (67.7) | 0.030 |
| Number of HBOT sessions | 2 [1, 3] | 3 [1, 3] | 2 [1, 3] | 0.001 |
| Length of hospital stay (days) | 1 [0, 3] | 1 [0, 3] | 1 [0, 2] | 0.001 |
| ICU stay (days) | 33 (4.3) | 26 (5.6) | 7 (2.4) | 0.035 |

Continuous data are expressed as median (25^th^ and 75^th^ percentiles); categorical data are presented as frequency (proportion).

**Supplementary Table 2**. Demographic and clinical data of patients with and without ECG recording

| Variables | Total  (*N* = 466) | With ECG recording  (*n* = 323) | Without ECG recording  (*n* = 143) | *P* value |
| --- | --- | --- | --- | --- |
| Male gender | 230 (49.4) | 148 (45.8) | 82 (57.3) | 0.022 |
| Age (years) | 33 [22, 45] | 35 [25, 46] | 25 [10, 42] | <0.001 |
| Psychiatric history | 82 (17.6) | 62 (19.2) | 20 (14.0) | 0.173 |
| Pulse, beats/min | 100 [84, 114] | 98 [84, 112] | 103 [85, 121] | 0.044 |
| Pulse > 100 beats/min | 216 (46.4) | 145 (44.9) | 71 (49.7) | <0.001 |
| Glasgow Coma Score (GCS) | 15 [14, 15] | 15 [14, 15] | 15 [11, 15] | 0.132 |
| GCS less than 9 | 62 (13.3) | 38 (11.8) | 24 (16.8) | 0.141 |
| Triage scale |  |  |  | <0.001 |
| 1, resuscitation | 74 (15.9) | 44 (13.6) | 30 (21.0) |  |
| 2, emergent | 290 (62.2) | 223 (69.0) | 67 (46.9) |  |
| 3, urgent | 94 (20.2) | 54 (16.7) | 40 (28.0) |  |
| 4/5, less urgent/not urgent | 8 (1.7) | 2 (0.6) | 6 (4.2) |  |
| Transferred from outside institution | 223 (47.9) | 157 (48.6) | 66 (46.2) | 0.625 |
| Attempted suicide | 190 (40.8) | 135 (41.8) | 55 (38.5) | 0.499 |
| Source of CO poisoning |  |  |  | 0.576 |
| Charcoal burning | 196 (42.1) | 134 (41.5) | 62 (43.4) |  |
| Water heater incomplete combustion or incorrect use of furnace | 238 (51.1) | 169 (52.3) | 69 (48.3) |  |
| Other | 32 (6.9) | 20 (6.2) | 12 (8.4) |  |
| Concomitant use with tranquilizer | 63 (13.5) | 49 (15.2) | 14 (9.8) | 0.117 |
| Transient loss of consciousness | 292 (62.7) | 202 (62.5) | 90 (62.9) | 0.935 |
| Duration of loss of consciousness |  |  |  | <0.001 |
| < 6 hours | 210 (45.1) | 152 (47.1) | 58 (40.6) |  |
| 6-12 hours | 28 (6.0) | 25 (7.7) | 3 (2.1) |  |
| 13-24 hours | 20 (4.3) | 11 (3.4) | 9 (6.3) |  |
| > 24 hours | 16 (3.4) | 10 (3.1) | 1. (4.2) |  |
| > 48 hours | 18 (3.9) | 4 (1.2) | 14 (9.8) |  |
| Leukocytosis | 245 (52.6) | 179 (55.4) | 66 (46.2) | 0.024 |
| Metabolic acidosis | 67 (14.4) | 49 (15.2) | 18 (12.6) | 0.306 |
| COHb, % | 10.1 [4.0, 22.6] | 10.1 [4.0, 21.3] | 10.6 [4.2, 25.4] | 0.408 |
| Troponin I, ng/mL | 0.20 [0.01, 1.83] | 0.31 [0.01, 2.10] | 0.10 [0.01, 1.30] | 0.056 |
| Evidence of myocardial injury | 64 (13.7) | 50 (15.5) | 14 (9.8) | <0.001 |
| Time from CO exposure to ED (hours) | 4.0 [2.5, 8.0] | 4.5 [2.5, 8.0] | 4.0 [2.5, 10.0] | 0.745 |
| Time from CO exposure to ECG recording (hours) | NA | 5.1 [3.1, 8.5] | NA | NA |
| QTc prolongation | 149 (32.0) | 137 (42.4) | 12 (8.4) | <0.001 |
| Brain CT imaging study at first medical institution | 131 (28.1) | 88 (27.2) | 43 (30.1) | 0.532 |
| HBOT | 279 (59.9) | 198 (61.3) | 81 (56.6) | 0.344 |
| Number of HBOT sessions | 3 [1, 3] | 3 [1, 3] | 3 [1, 3] | 0.867 |
| Length of hospital stay (days) | 1 [0, 3] | 1 [0, 4] | 1 [0, 3] | 0.240 |
| ICU stay (days) | 26 (5.6) | 22 (6.8) | 4 (2.8) | 0.082 |
| Lucid interval, duration from exposure to DNS (days) | 10 [7, 24] | 10 [6, 24] | 11 [8, 23] | 0.901 |

Continuous data are expressed as median (25^th^ and 75^th^ percentiles); categorical data are presented as frequency (proportion).
